# Active Ingredients in Digital Cognitive Interventions: Integrating Dismantling Designs With Mechanistic Neuroscience

**DOI:** 10.2196/94246

**Published:** 2026-07-14

**Authors:** Sarah Shizuko Morimoto, Cutter Augustus Lindbergh, Alexander Conley, Dusti R Jones, David C Steffens

**Affiliations:** 1 Division of Health Systems Innovation and Research, Department of Population Health Sciences, University of Utah, Williams Building, 295 Chipeta Way, Salt Lake City, UT, 84108, United States, 1-801-587-1288; 2 Department of Psychiatry, UConn Health, Farmington, CT, United States; 3 Department of Psychiatry, Center for Cognitive Medicine, Vanderbilt University Medical Center, Nashville, TN, United States; 4 Department of Family and Preventative Medicine, University of Utah, Salt Lake City, UT, United States

**Keywords:** digital, cognition, dismantling, active ingredients, cognitive remediation

## Abstract

Digital cognitive interventions (DCIs) have emerged as scalable approaches for treating cognitive dysfunction across psychiatric, neurological, and aging populations. Despite growing evidence of efficacy, little is known about which intervention components drive therapeutic effects or through which neurocognitive mechanisms they operate. As a result, null findings are often difficult to interpret, making it unclear whether interventions failed to engage their intended targets, or whether the targets themselves are not causally related to meaningful outcomes. This limits intervention refinement, comparative evaluation, and precision personalization. Here, we argue that DCI research should shift from broad efficacy testing toward mechanistic trials designed to identify active ingredients—the intervention components responsible for engaging prespecified neurocognitive targets and producing clinically meaningful benefits. We propose adapting dismantling design methodology from psychotherapy research in order to integrate Research Domain Criteria constructs, mechanistic neuroscience, and high-resolution digital behavioral data to identify factors driving cognitive and functional outcomes. This approach aligns with the National Institute of Mental Health experimental therapeutics framework by explicitly linking target specification and target engagement with downstream clinical and functional outcomes. Mechanistic dismantling trials can determine whether specific DCI features, including adaptive difficulty, reward schedules, feedback contingencies, task variability, cognitive targets, and human support, are necessary, sufficient, or synergistic for engaging neural circuitry and producing durable and clinically meaningful transfer. Beyond optimizing intervention design, such studies may transform null or negative trials into mechanistically interpretable findings, while clarifying disease mechanisms and supporting the development of personalized, optimized, and usable DCIs.

## Introduction

### Role of Cognitive Deficits in Psychiatric Disorders

The common and persistent cognitive deficits that accompany psychiatric disease represent a major, yet underrecognized, source of global clinical, economic, and social burden across disorders [1], with many major psychiatric disorders ranking among the top causes of long-term functional impairment [2]. Furthermore, cognitive impairment is a core and transdiagnostic feature, encompassing deficits in attention, processing speed, working memory, executive functioning, and learning [1,3]. Data suggest that cognitive deficits are often present early in illness onset, are observable across childhood, adulthood, and older age, and persist despite psychiatric symptom remission [3-6]. In schizophrenia and bipolar disorder, impairments in executive functioning and verbal memory can be characterized as “moderate to severe” and may be detectable prior to first episode as well as during euthymic mood states [7]. In unipolar mood, anxiety, and trauma-related disorders, deficits in cognitive control and processing speed reliably persist following clinical recovery [1,3,8]. Importantly, the magnitude of cognitive impairment shows only weak-to-modest correlations with symptom severity, supporting the view that cognition represents a partially *independent dimension of psychopath*o*logy* [9-11]. Cognitive deficits are robust predictors of real-world functioning—including employment, social functioning, and independent living—and are a primary driver of psychosocial disability across depression and transdiagnostic psychiatric populations [12-14].

Among cognitive domains, deficits in executive function or cognitive control exert particularly important prognostic and treatment-modifying effects across psychiatric disorders [15,16]. Baseline executive impairment predicts poorer response to pharmacologic and psychotherapeutic interventions, slower and less complete functional recovery, increased risk of relapse and chronicity, higher all-cause mortality, and elevated suicide risk [6,15-22]. Despite these effects, standard psychiatric treatments, including antidepressants, antipsychotics, mood stabilizers, and evidence-based psychotherapies, primarily target emotional or behavioral symptoms and do not reliably improve cognition [23,24]. Large randomized trials and network meta-analyses indicate that, with few exceptions, commonly prescribed treatments yield minimal or no clinically meaningful improvement in objective cognitive outcomes. Consequently, many patients achieve symptomatic remission while continuing to experience persistent cognitive and functional disability, contributing to the well-documented gap between clinical (symptomatic) recovery and real-world (functional) recovery. Collectively, these findings identify cognitive impairment as a central, enduring, and functionally consequential determinant of disability across psychiatric conditions [15]. The transdiagnostic nature of executive functioning deficits, their disproportionate contribution to economic and social burden, and their limited responsiveness to existing treatments highlight cognition as a critical unmet target for clinical intervention and public health impact [15,16].

Neuroplasticity-based digital cognitive interventions (DCIs) have emerged as brief, safe, accessible, and scalable adjunctive treatments that provide structured training to ameliorate cognitive deficits in psychiatric populations. (*Neuroplasticity-based DCIs are computerized training programs designed to drive experience-dependent changes in specific neural systems through repeated, targeted practice, often with adaptive difficulty and immediate feedback. Treatment effects may reflect neural target engagement rather than simple task repetition or general engagement.*). We and others have demonstrated that DCIs targeting cognitive control functions, even in the aging brain, can improve cognitive performance (eg, inhibition, semantic clustering, and executive organization) and reduce depressive symptoms in individuals with major depressive disorder (MDD) [25-28]. These findings support the hypothesis that engagement of cognitive control mechanisms may yield downstream clinical benefits in MDD [29]. However, despite growing interest, the field of DCIs has reached a critical inflection point: the neurobiological mechanisms through which DCIs exert their effects remain poorly specified, inadequately tested, and frequently confounded with the more general experience of treatment engagement itself.

**Figure 1.**
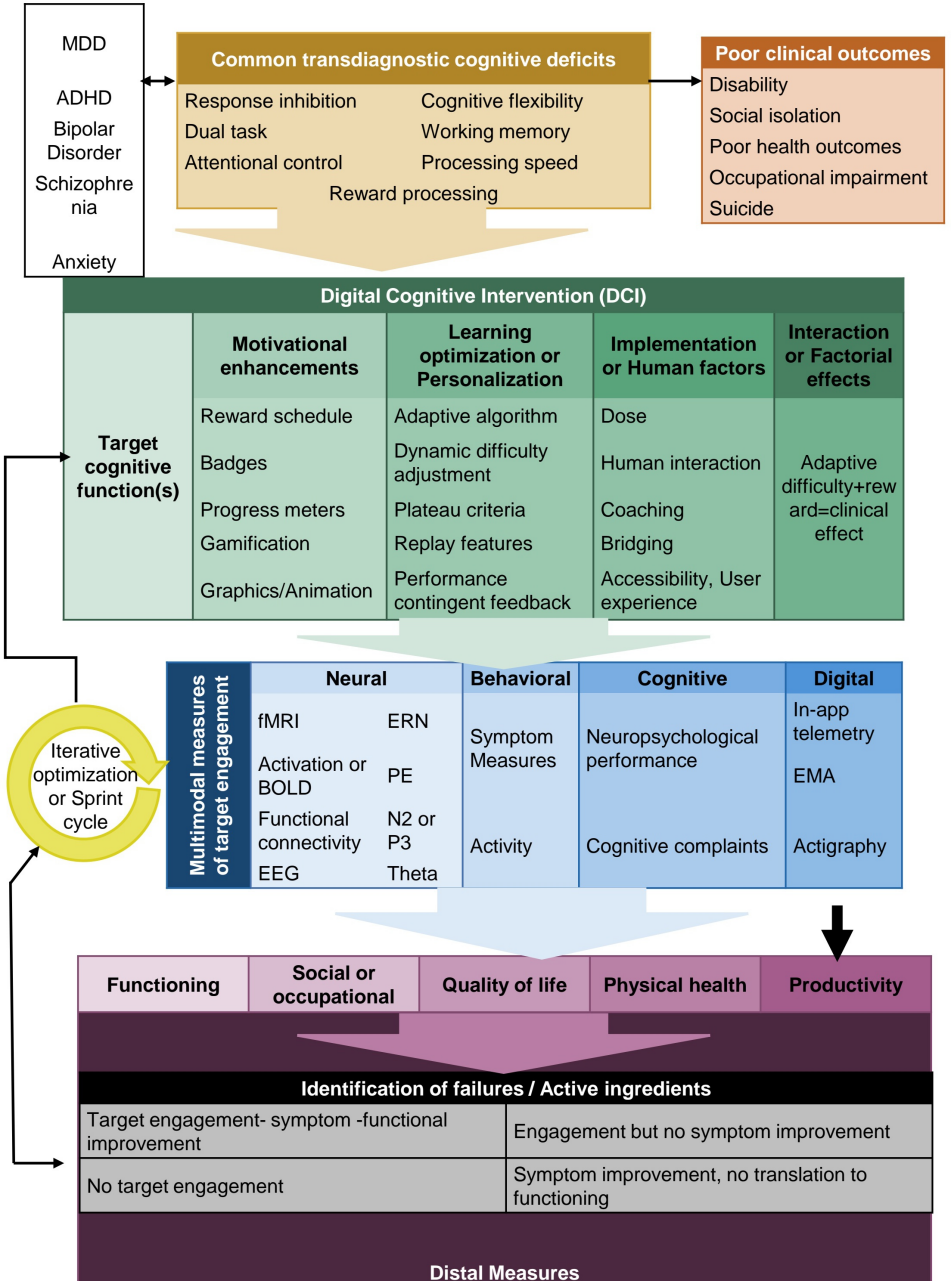
Schematic of the proposed multimodal or multimethod approach to investigating the active ingredients of digital cognitive interventions (DCIs). ADHD: attention-deficit/hyperactivity disorder; BOLD: blood-oxygen-level-dependent; EEG: electroencephalography; EMA: ecological momentary assessment; ERN: error-related negativity; fMRI: functional magnetic resonance imaging; MDD: major depressive disorder; N2: maximum negative deflection; P3: maximum positive deflection; PE: error positivity; QOL: quality of life; sx: symptoms; UX: user interface.

This review synthesizes emerging evidence on DCIs through a multimethod, multimodal lens to clarify mechanisms of action and optimize intervention design (Figure 1). We review the current landscape of DCIs and highlight key design features hypothesized to drive neuroplasticity, including adaptivity, task specificity, and engagement strategies. We suggest that dismantling methodology, adapted from early studies of psychotherapeutic interventions and applied to DCI clinical trials, may isolate the mechanisms that do and do not drive neuroplasticity. By mapping these elements onto National Institute of Mental Health (NIMH) Research Domain Criteria (RDoC) constructs, we suggest candidate operationalizations of mechanistic hypotheses (Table 1). Building on this, we discuss experimental approaches, such as dismantling designs and adaptive trials, that can isolate active ingredients. Finally, we propose an integrated framework that combines behavioral, neural, and clinical endpoints to guide the rigorous review, development, and investigation of mechanistically informed, precision-targeted DCIs.

**Table 1.** Candidate active ingredients for DCIs^a^ suitable for DCI dismantling studies, their RDoC^b^-associated “targets,” candidate mechanistic measurements, and hypothesized outcomes.

DCI element	RDoC construct	Target domain	Target marker and modality	Example hypotheses^c^
Dual task coordination	Cognitive systems	Cognitive control (goal selection or maintenance); attention	fMRI^d^: dorsal attention network; ACC^e^; SMA^f^; cerebellum; frontoparietal EEG^g^ or MEG^h^: ERN^i^ or P3^j^; frontoparietal coherence; switch positivity	Improvements in cognitive flexibility associated with enhanced dorsal attention network connectivity during switch vs repeat tasks (fMRI) and larger centroparietal switch positivity during shifts between tasks (EEG or MEG) following DCI.
Response inhibition tasks	Cognitive systems	Cognitive control (inhibition or suppression); performance monitoring	fMRI: right IFG^k^, pre-SMA, STN^l^; ACC activation patterns EEG or MEG: sensorimotor beta power; N2^m^ or P3; LRP [30,31]	Improved inhibition or suppression associated with larger N2 or P3 and reduced LRP^n^ for no-go compared to go stimuli for active vs control training groups. Also, greater ACC connectivity within executive control network during inhibition tasks following DCI.
Speed-of-processing escalations	Cognitive systems; arousal or regulatory systems	Attention (orienting or sustained); arousal	Pupillometry: LC-NE^o^ system (pupil-linked arousal) EEG or MEG: thalamocortical pacing; visual cortex timing; alpha suppression	Improved sustained attention following DCI is evidenced by reduced pupil dilation during DCI sessions. Also, increased thalamocortical connectivity indicated by greater theta-gamma coupling for relevant vs irrelevant stimuli post-DCI.
Gamification (badges, narratives)	Positive valence systems; social processes	Reward responsiveness or learning; social communication (engagement)	fMRI: VS^p^, vmPFC^q^; dopaminergic signals; autonomic arousal EEG or MEG: feedback-related negativity [32,33]	Increased activity in the VS and vmPFC for positive vs neutral stimuli post-DCI. Greater ERN observed for trials with high vs low reward value for active vs control DCI.
Adaptive difficulty algorithms	Cognitive systems; arousal or regulatory systems	Performance monitoring; cognitive control (goal updating)	fMRI: ACC (conflict or error); dlPFC^r^; dynamic FC^s^ EEG or MEG: phasic pupil or ERN; frontal midline theta [34]	Increased frontal midline theta during high vs low conflict trials following DCI. Increased within-network connectivity of the executive control network during conflict tasks at post-DCI compared to pre-DCI.
Reward schedules (points, streaks)	Positive valence systems	Reward valuation; reward learning (model-free or model-based)	fMRI: VS, dorsal striatum; OFC^t^; FC reward network ECG or PPG^u^: HRV	Improved connectivity in regions within the reward network for active vs control DCI. Higher HRV^v^ in participants associated with increased levels of reward training across DCI.
Plateau and graduation criteria	Cognitive systems	Performance monitoring; preparation; goal maintenance	fMRI: ACC; dlPFC; learning-curve signatures EEG or MEG: higher theta ITPC^w^ [35]	Participants with faster-level completion show increased connectivity between ACC and dlPFC compared to participants with slower completion. Increased theta ITPC associated with the duration of training on DCI.
Implementation strategies (eg, “if-then” plans; strategy coaching)	Cognitive systems; social processes	Cognitive control (goal selection or maintenance); perception and understanding of self (metacognition)	fMRI: dlPFC-vmPFC coupling; frontoparietal-DMN^x^ interactions EEG or MEG: metacognitive ERPs^y^; connectivity shifts; LRP, parietal P300	Stronger connectivity within the prefrontal cortex observed for active vs control DCI. Improved reactive control processing, observed in greater parietal P300 and smaller Gratton-dip for noninformative stimuli in the LRP following DCI.
Sensory stimulation (sounds, art, color)	Perception; arousal or regulatory systems	Auditory or visual perception [36]; arousal	fMRI: modality-specific cortices; salience network LC-NE arousal EEG or MEG: alpha or gamma gain mismatch negativity, P100, N100, N2pc^z^	Greater internetwork connectivity for salience network in participants receiving active vs control DCI. Larger N2pc amplitudes following DCI for task-relevant vs irrelevant stimuli.
Human or computer interactions (guidance, chat, coaching, alliance)	Social processes; arousal or regulatory systems	Social communication; affiliation or attachment; arousal	fMRI: temporoparietal junction; mPFC^aa^ (mentalizing) EEG or MEG: P1, N170, microstates	Increased activation of the mPFC following coached DCI session vs noncoached session. Alterations in self-referential EEG microstates C and D following active vs control DCI.

a DCI: digital cognitive intervention.

b RDoC: Research Domain Criteria.

c Examples are provided of the types of hypotheses that may be expected in dismantling studies. However, it should be noted that this is a nonexhaustive list, and the precise hypothesis (eg, target network engaged and directionality of effects) will depend upon the clinical population and the context of the investigation.

d fMRI: functional magnetic resonance imaging.

e ACC: anterior cingulate cortex.

f SMA: supplementary motor area.

g EEG: electroencephalography.

h MEG: magnetoencephalography.

i ERN: error-related negativity.

j P3: maximum positive deflection.

k IFG: inferior frontal gyrus.

l STN: subthalamic nucleus.

m N2: maximum negative deflection.

n LRP: lateralized readiness potentials.

o LC-NE: locus coeruleus-norepinephrine system.

p VS: ventral striatum.

q vmPFC: ventromedial prefrontal cortex.

r dlPFC: dorsolateral prefrontal cortex.

s FC: functional connectivity.

t OFC: orbitofrontal cortex.

u ECG: electrocardiography.

v HRV: heart rate variability.

w ITPC: intertrial phase clustering.

x DMN: default mode network.

y ERP: event-related potential.

z N2pc: N2 posterior contralateral.

aa mPFC: medial prefrontal cortex.

The inability to disentangle engagement from mechanism represents a major barrier to scientific progress. Without isolating the “active ingredients” of DCIs, it is difficult to interpret null findings, optimize intervention design, or personalize treatment. Most DCI studies operationalize engagement using objective usage metrics (eg, number of logins, duration of use, and task completion) and examine associations between these metrics and clinical outcomes [37]. However, engagement is rarely manipulated independently of intervention content, nor is it experimentally dissociated from the hypothesized therapeutic mechanism. As a result, observed improvements may reflect nonspecific effects of sustained engagement, novelty, or expectancy rather than selective modulation of a theoretically defined cognitive process. This limitation substantially constrains causal inference and impedes the development of mechanistically precise and generalizable interventions; thus, underlying mechanisms related to clinical improvement with DCIs remain unclear [38-40].

### Active Ingredient Definition

For clarity, we define *active ingredients* as the discrete features of a DCI that are hypothesized to causally engage neural mechanisms. This contrasts with nonspecific aspects such as mere time-on-task or expectancy. Identifying these ingredients requires isolating them experimentally. For example, one could compare a full-feature DCI (with rewards and variable difficulty) to a version that is identical except for rewards. Differences in brain and behavioral outcomes between conditions would then implicate reward as an active ingredient.

## Methodological Limitations of Current Active Control Conditions

Efforts to address nonspecific effects through the inclusion of “active control” conditions have, to date, been largely insufficient [41]. Another limitation is the lack of standardized, theory-driven approaches for control conditions [42]. Clinical trials of DCI efficacy often use controls that include commercial games, educational applications, or expectancy-matched cognitive tasks, all matched on exposure duration with the active DCI. Many used comparator interventions designed to engage overlapping cognitive processes, and most differ substantially in adaptive difficulty, reward deployment, performance-based feedback, motivational salience, or a combination of these elements [41]. Without controls that are explicitly designed to not promote mechanistic hypotheses, it remains unclear whether interventions engage their intended targets or simply enhance engagement.

The NIMH experimental therapeutics (ET) framework emphasizes the identification and engagement of specific, neurobiological theory-driven targets, with the explicit goal of determining whether modulation of those targets produces meaningful clinical change. In practice, ET terminology is often applied to DCIs that are designed to be optimized for symptom reduction rather than for target engagement. Engagement metrics are frequently treated as proxies for mechanisms, rather than as prerequisites or moderators of mechanistic change. An important methodological critique of this practice is that it slows progress by generating signals of efficacy without advancing mechanistic understanding of how or why interventions work [43].

Addressing these limitations is of high significance for both basic and translational science. Without rigorous tests of mechanism, the field cannot reliably optimize intervention components, predict individual response, or translate DCIs into real-world clinical benefits. Moreover, failure to adhere to ET principles limits the interpretability of null findings, making it unclear whether an intervention failed due to inadequate target engagement or because the target itself is not causally related to outcomes. Psychotherapy dismantling studies emerged in the 1970s and 1980s as part of broader component analysis efforts in clinical psychology, aiming to isolate the specific techniques within multicomponent behavioral interventions that drive therapeutic change [44].

Here we advocate for the design of trials of DCIs to address this critical gap by prioritizing mechanistic specificity over broad efficacy and by using control conditions capable of supporting strong causal inference. What we propose may be a costly and time-consuming effort; however, aligning DCI research with ET principles has the potential to substantially advance the rigor, reproducibility, and translational impact of the field, fundamentally shifting intervention development toward parsimonious, mechanism-informed designs.

## The Case for Mechanistic Research in DCIs

### Reward Schedules as a DCI Element in Mechanistic Dismantling With Clinical Neuroscience

Little is known about *how* reward contingencies, reward schedules, and reward deployment—core features of digital design—engage well-characterized dopaminergic systems implicated in motivation, learning, and cognitive control [45,46]. From a neuroscience perspective, dopamine is not only central to reward processing but also plays critical roles in both synaptic plasticity and the updating of cognitive control processes in the prefrontal cortex [47,48]. Dopamine may be particularly important for strategic processing under conditions of uncertainty and adaptive demand [49,50]. Performance-contingent and variably deployed rewards modulate phasic dopamine signaling within mesolimbic and frontostriatal circuits, reinforcing action-outcome associations and regulating learning rates and effort allocation [49,51]. Consequently, reward schedules embedded within cognitively demanding tasks may influence both adherence to the tasks and the efficiency of cognitive skill acquisition and generalization [52]. For example, when behavior modification is the desired outcome, devices such as Fitbit (Google) or Apple Watches deliver many and varied real-time metrics to reinforce users’ awareness of progress toward goals, while continuous performance monitoring enables immediate cues for action and opportunities for proximate and frequent rewards [53,54].

Disrupted reward processing, related to blunted striatal responsivity and reduced dopaminergic signaling, is prevalent in many psychiatric disorders and is closely linked to anhedonia, diminished motivation, impaired cognitive flexibility, and poor functional outcomes [55,56]. These abnormalities suggest that reward deployment may not act only as a critical *facilitator* of engagement and learning but may also represent an “active ingredient” of DCIs. Reward exposure alone does not produce durable cognitive or clinical improvement in the absence of task demands that directly engage targeted neural systems (such as cognitive control), suggesting that motivation without mechanism is insufficient for sustained change [57,58]. Consistent with this, commercial and “serious” video games reliably improve task-specific perceptual and attentional skills, yet these gains rarely transfer to broader cognitive or functional outcomes [59,60]. This limited transfer likely reflects inadequate engagement of higher-order cognitive control *with* rewarded gameplay, rather than insufficient task engagement or persistence [60].

### Controlled Tasks to Complex Systems: How Reward-Learning Coupling Differs in DCIs

#### Overview

Decades of cognitive neuroscience research show that reward contingencies shape learning through dopamine-mediated mechanisms, particularly via reward prediction error signals that encode discrepancies between expected and actual outcomes and drive synaptic plasticity [50,61]. Critically, laboratory studies demonstrate that reward alone is insufficient; learning requires active, task-contingent engagement, with immediate, performance-contingent feedback producing the strongest neural error signals and behavioral gains [48,50,51,61]. Both laboratory tasks and DCIs rely on shared neurobiological mechanisms—dopamine-mediated modulation of frontostriatal plasticity supporting learning, attention, and cognitive control [50,51,61]. However, they differ in the following 3 key ways.

#### Complexity

DCIs implement reward principles within multicomponent, gamified environments that integrate points, levels, and narratives [62]. Although engaging tasks (eg, video games) can elicit dopaminergic responses [45,63], DCI rewards are typically abstract (eg, points and badges) rather than primary or monetary, potentially engaging reward systems differently. Gamification may enhance motivation [62] but also imposes additional cognitive demands (eg, navigation, narrative tracking) that are orthogonal to the target training mechanism.

#### Timing

DCIs operate over extended timescales, often incorporating adaptive difficulty across weeks or longer. This resembles spaced learning processes that support consolidation and more durable, transferable learning [64].

#### Context

DCIs are influenced by psychological and contextual factors largely absent in laboratory settings. User expectations, motivation, and engagement can substantially shape outcomes [62]. Notably, placebo-controlled studies show that beliefs about cognitive training efficacy can independently enhance performance [65].

RDoC proposes both cognitive control and reward learning as dimensional, transdiagnostic constructs instantiated in well-characterized neural circuitry. Mapping DCI components onto RDoC domains, particularly Cognitive Systems and Positive Valence Systems, may provide a defined framework for ensuring that intervention elements are explicitly linked to measurable targets.

## Dismantling Studies as a Model for the Future of DCI Research

Dismantling studies provide a methodological framework by which the relative efficacy of DCI components can be systematically compared, often via randomized controlled designs [66]. Applying dismantling logic to DCIs would allow researchers to (1) test the necessity and sufficiency of individual cognitive paradigms; (2) identify minimal effective doses; (3) determine whether changes arise from specific cognitive mechanisms (eg, updating and set shifting) versus nonspecific factors such as engagement, expectancy, or gamification [65,67]; or (4) examine synergistic combinations of elements [67]. These studies will allow for the optimization of current DCIs and will aid in developing a component “active ingredient” library that may enable head-to-head comparisons of DCIs.

Rather than viewing dismantling strictly as “remove X and see what happens,” intervention developers may characterize their methods by manipulating specific RDoC dimensions. For example, removing or altering gamified reward contingencies may allow for testing the role of positive valence system reward responsiveness on intervention outcomes. Alternatively, removing adaptive difficulty within DCIs for interference resolution may interrogate the role of the cognitive systems domain. Characterizing intervention elements by RDoC constructs elevates dismantling methodology from simple component testing to potentially transdiagnostic construct-specific hypothesis testing.

## Integrating Mechanistic Neuroscience and Dismantling Designs

Integrating mechanistic neuroscience into dismantling studies enables direct tests of whether specific DCI components engage intended neural targets rather than producing nonspecific behavioral effects [41]. Consistent with the RDoC framework, this approach supports multilevel measurement across behavior, physiology, and neural circuits. Neuroimaging and electrophysiological studies demonstrate that DCIs can be differentiated at the neural level even when surface-level engagement appears similar [28].

In MDD, for example, our CoNECCT (Computerized Neuroplasticity Enhancing Cognitive Control Training) intervention has been associated with changes in frontoparietal and cingulo-opercular control networks, including increased medial prefrontal cortex-insula functional connectivity linked to reduced social apathy, and increased dorsal anterior cingulate cortex activation during emotion regulation associated with symptom improvement [68] More broadly, functional magnetic resonance imaging (fMRI) studies indicate that adaptive, performance-contingent task demands preferentially engage executive control circuitry, whereas nonadaptive versions do not, despite similar exposure [69]. Complementary electroencephalography (EEG) findings show component-sensitive changes in neural dynamics, such as frontal midline theta and alpha power, that track cognitive control, effort allocation, and learning, and distinguish active training from passive or sham conditions [70,71]. In other disorders, including schizophrenia and attention-deficit/hyperactivity disorder, dismantling-informed studies demonstrate that adaptive difficulty and feedback selectively modulate frontostriatal coupling and reward prediction error signaling, supporting a mechanistic role for these features [72-74]. Together, fMRI and EEG provide complementary insight: fMRI identifies changes in activation and connectivity within target circuits (eg, frontoparietal control and frontostriatal reward systems), while EEG captures temporally precise markers of cognitive control and reinforcement learning, including frontal midline theta, feedback-related negativity, and N2 (maximum negative deflection) or P3 (maximum positive deflection) dynamics [75,76].

At the same time, integrating neuroimaging into DCI studies introduces important considerations, including participant burden, motion and fatigue artifacts, cost, and the limited ecological validity of scanner-based tasks. Pre- or post designs may also capture exposure or practice effects rather than component-specific mechanisms. Accordingly, neuroimaging should be anchored to prespecified mechanistic hypotheses and interpreted within a dismantling and ET framework [72,73].

## Designing Dismantling-Inspired Mechanistic Studies for DCIs

### Component Classification

Dismantling design requires precise classification of intervention elements. Components of DCIs may include the cognitive task itself (eg, working memory and inhibitory control), pacing or scheduling features (eg, adaptive difficulty, dose, and duration), and reward structures (eg, points and badges). Disentangling these components allows investigators to test which features drive neural and behavioral improvements, rather than improvements due to engagement or placebo effects.

### Mechanistic Hypothesis

Each intervention component selected for systematic removal within the dismantling design should be explicitly linked to a priori mechanistic hypotheses and prespecified, clinically relevant downstream outcomes. For each component, hypotheses should specify (1) the targeted neural and cognitive systems, (2) the expected form of neuroplastic change within those systems, and (3) the predicted behavioral or cognitive consequences. It is hypothesized that component removal will result in attenuated, altered, or absent effects on these mechanistic and clinical outcomes, thereby enabling causal inferences about the hypothesized active ingredients. Primary outcomes in dismantling studies of DCIs should prioritize direct assessment of hypothesized neural and cognitive mechanisms relevant to the target disorder, rather than relying exclusively on global symptom measures. Neurobiological (eg, network-level activation and connectivity) and cognitive (eg, executive control, learning efficiency) outcomes should be complemented by gold-standard clinical assessments to establish clinical relevance.

For example, the dynamic difficulty adjustment is hypothesized to engage frontoparietal executive control networks by continuously increasing cognitive demands, thereby recruiting overlapping neuromodulatory systems and reducing reliance on automatized or rote responding [77]. Sustained engagement of these systems is expected to promote experience-dependent plasticity within cognitive control circuits [78], resulting in measurable improvements in cognitive flexibility, adaptive control, and related disorder-relevant cognitive functions [79]. Removal of adaptive difficulty would be predicted to yield reduced engagement of these networks, diminished neuroplastic change, weaker cognitive effects, and functional outcomes. Differential outcomes following the removal or alteration of this component may then provide preliminary evidence for its mechanistic role.

### Dose

A critical, often overlooked, parameter in DCIs is the dose of cognitive training, that is, the amount and intensity of practice required to induce meaningful plasticity. Unlike pharmacologic treatments, optimal dosing for DCIs remains poorly specified. Emerging evidence suggests a nonlinear dose-response relationship and further indicates that both insufficient and excessive training may be suboptimal [80]. This pattern is consistent with neuroplasticity models, which show that repeated, paced practice over weeks is required for durable circuit-level change, whereas brief or overly intensive exposure may yield only transient effects [81]. Accordingly, dose should be treated as a core active ingredient in dismantling studies. Systematically varying session length, frequency, and total duration, and including lower-dose comparison arms, can help identify the minimally effective dose needed to produce neural and cognitive change, distinguishing true component effects from differences in exposure [82]. Administering key outcome measures at regular intervals throughout the intervention, rather than solely at the end of the treatment, can also help quantify dose-response curves.

### In-App Telemetry

In addition, DCIs uniquely enable the continuous collection of high-resolution digital behavioral data, offering the opportunity to identify novel digital behavioral markers of mechanistic engagement and treatment response. In-game metrics such as adaptive performance slopes, trial-by-trial error monitoring, and intraindividual variability in response times may index dynamic learning processes (ie, “learning to learn”) and real-time deployment of cognitive control. When integrated with neuroimaging and standardized cognitive measures, these digital markers may provide sensitive, scalable indicators of target engagement as well as clinical and cognitive change. Together, this multimodal mechanistic approach will allow investigators to distinguish neural and cognitive effects attributable to specific intervention components from nonspecific influences such as general practice effects or task exposure.

### Dismantling Randomized Controlled Trial Structure

Dismantling-inspired designs use randomized controlled trial structures that systematically compare the full intervention to versions in which specific components are removed. For example, a 2×2 factorial design can independently manipulate adaptive difficulty and feedback, yielding 4 experimental trial conditions: full intervention with adaptive difficulty and feedback, adaptive difficulty only, feedback only, and an active control matched for time and engagement but without adaptive difficulty or feedback. This design, combined with pretests and posttests, allows estimation of the main effects of each component and their interactions, paralleling approaches used in psychotherapy dismantling studies [83] and recent DCI trials in schizophrenia [72].

### Mediation Modeling

To establish candidate causal pathways between DCI components and clinical improvement, changes in RDoC-defined constructs hypothesized that mechanisms can be tested as mediators of long-term clinical outcomes. By anchoring intervention elements in RDoC rather than traditional diagnostic categories (eg, MDD only), the dismantling framework tests whether targeting RDoC constructs changes core dysfunctional processes that *cut across multiple disorders* (eg, cognitive control deficits in depression, anxiety, and attention-deficit/hyperactivity disorder). For instance, repeated-measures or longitudinal mediation models can examine whether DCI-induced increases in dorsolateral prefrontal cortex-anterior cingulate cortex functional connectivity, frontal midline theta, or cognitive control task performance from baseline explain subsequent reductions in depressive symptoms or functional impairment. Mixed effects models allow the decomposition of within-participant (change over time within an individual) and between-participant (individual differences from the group, sample, or population) effects, enabling separation of temporal sequences from stable processes, which are critical for establishing that mechanistic change *precedes* clinical benefit [84,85]. Structural equation modeling can also be applied to simultaneously test multiple candidate mediators (eg, cognitive control vs reward learning or functional connectivity) to identify which mechanisms are most directly responsible for observed improvements [86]. Prior studies in schizophrenia and depression have successfully applied these approaches, demonstrating that training-induced increases in frontostriatal connectivity or executive control performance mediate improvements in functional outcomes and mood, providing empirical support for the mechanism-to-clinical benefit pathway endorsed by the NIMH’s RDoC criteria [28,72,73].

### Moderation Testing

Identifying moderators of DCI efficacy may enable both within-app personalization and mechanistic specificity. Factors such as age, baseline cognitive performance, symptom severity, genetic polymorphisms, structural or functional imaging, or inflammatory biomarkers can influence both the degree to which participants engage with specific intervention components and the neuroplasticity induced by engaging with DCIs. For example, in late-life depression, individuals with greater baseline cognitive control deficits may show differential recruitment of prefrontal circuits in response to adaptive cognitive training, moderating the magnitude of cognitive gains [28]. In dismantling trials, moderation analyses are typically conducted via interaction terms in mixed effects models or Bayesian hierarchical models, allowing simultaneous estimation of within- and between-individual effects (ie, individual-level and group-level effects, respectively). By integrating baseline neurocognitive and biological markers, researchers can identify subgroups most likely to benefit from particular components (eg, reward modulation, adaptive difficulty, or plateau and graduation criteria).

Such dismantling-informed designs provide a mechanistic map of intervention efficacy, offering an evidence-based process for streamlining DCI by assessing whether removing specific components diminishes cognitive, neural, or clinical outcomes. For example, in the DCI CoNECCT [26,68], if removing adaptive difficulty reduces frontoparietal engagement and blunts cognitive gains, it could be classified as a core contributor, whereas reward function may primarily enhance adherence without directly driving neuroplasticity in target networks [68].

## Active Control Conditions and the Case for Dismantling DCIs

Trials of DCIs in psychiatric populations have used a variety of control and comparator conditions to distinguish specific treatment effects from nonspecific factors, such as engagement, expectation, or human interaction. Active control conditions often involve nonadaptive or low-demand versions of the same cognitive tasks, designed to match intervention participants for time, interface exposure, and motivation, but without specifically engaging the hypothesized neurocognitive mechanisms in as high a “dose.” For example, in computerized cognitive remediation for schizophrenia, Subramaniam et al [72] used a control condition comprising computer-based general knowledge games matched for engagement but lacking executive function demands. Similarly, in our trials, we implemented an active digital control consisting of computer exercises matched for duration and feedback but not specifically designed to drive neuroplastic changes in cognitive control networks [25,26,68]. Sham interventions provide superficially similar tasks without adaptive difficulty, controlling for placebo and novelty effects, while treatment-as-usual or waitlist controls assess intervention effects relative to standard care. While these comparators help establish efficacy, they do not clarify which *specific* elements of the intervention drive cognitive or clinical change. The dismantling framework, by contrast, systematically isolates individual components—such as dynamic difficulty adjustment, reward contingencies, or feedback—and compares full versus component-removed versions. This approach allows investigators to test whether each element engages its hypothesized neural and cognitive mechanisms, providing clearer evidence for causal pathways from intervention to outcome.

## DCIs as Tools for Causal Neuroscience

We have proposed that DCIs act as both an intervention for and a probe of the neurobiology of their end users. For example, CoNECCT uses algorithms to dynamically adapt to the real-time cognitive status of the user along 5 different game components, including accuracy in processing speed, inhibitory control, and cognitive flexibility [25]. This design allows for parametric manipulation of cognitive load across multiple functions, reward schedules, frustration tolerance, learning potential, and response to performance-based feedback. Furthermore, DCIs can be designed to record every keystroke to the millisecond, allowing for cross-sectional and longitudinal cognitive assessment, and its relationship to clinical outcomes of interest in any population in which it is deployed. If identified, “active ingredients” can act as a dynamic probe of reward sensitivity, cognitive control, response to negative or positive feedback, among other factors, and can be compared between psychiatric disorders in terms of performance, plastic potential, and clinical impact. Thus, active ingredients not only inform why and how a particular DCI works but also shed light on specific disease mechanisms underlying cognitive and clinical impairments in the target population and across disorders.

## Limitations and Future Directions

While dismantling studies of DCI components hold considerable promise, important limitations and design challenges remain. Existing studies are often small, use short-term follow-up assessments, or focus on a single diagnosis or RDoC domain, limiting statistical power to detect component-specific effects and reducing transdiagnostic generalizability [39,43,62]. In practice, DCI elements likely interact in nonlinear and potentially synergistic ways. For example, adaptive difficulty may only produce neural gains when paired with meaningful, performance-contingent feedback; without feedback, users may disengage. Conversely, reward deployment may have little effect if tasks lack sufficient cognitive demand. Similarly, human support may enhance adherence and effective dose, indirectly enabling other components (eg, adaptive algorithms) to produce measurable neuroplastic change. These interdependencies complicate causal inference. Removing a component may inadvertently reduce overall “dose” (eg, time on task or effort), such that diminished neural or behavioral effects reflect reduced exposure rather than the absence of a specific active ingredient. Accordingly, dismantling designs should incorporate factorial structures or combination arms to capture interaction effects and carefully monitor adherence (eg, sessions completed and time on task). Statistical models should account for dose as a covariate to distinguish ingredient-driven from exposure-driven effects. Dose itself may also interact with component efficacy: a given element may appear inert at low exposure but become active at higher doses (or vice versa), suggesting the value of including intermediate dose conditions.

Additional complexities arise from temporal dynamics: neuroplastic changes induced during training may not translate immediately into clinical improvement. Follow-up assessments (eg, 3‐6 mo) are, therefore, critical to detect “sleeper effects,” where early neural target engagement predicts later functional or symptomatic gains. Longitudinal mediation models can clarify these pathways by testing whether early changes in neural or cognitive markers predict downstream functional outcomes. Conversely, the presence of robust neural change without subsequent clinical improvement is equally informative, suggesting that target engagement alone may be insufficient without additional mechanisms supporting generalization or real-world application.

Finally, individual differences in neural plasticity, reward sensitivity, and baseline cognitive capacity may moderate both engagement and response to specific components, underscoring the need for adequately powered moderation analyses. Careful consideration of these factors is essential for designing interpretable dismantling studies and for advancing a mechanistically precise science of DCIs.

## From Generic Nulls to Mechanistically Interpretable Nulls

Neuroscience-informed dismantling designs offer a critical opportunity to distinguish neural target engagement from clinically meaningful transfer. Such studies may reveal DCIs that achieve mechanistic success without functional impact; for example, demonstrating robust neural plasticity (eg, changes in connectivity or oscillatory dynamics) without corresponding improvements in real-world outcomes. This dissociation suggests that target engagement alone is insufficient for generalization and reframes negative trials as informative, helping to identify where the causal chain from intervention to outcome breaks down.

One potential interpretation is that measurable cognitive improvements lack the scaffolding needed for flexible application to the patients’ environment. Even when target networks show expected changes, improvements may remain transient or evident during training but fail to persist or generalize across contexts. High-resolution behavioral data can help detect such patterns, revealing steep within-task learning that does not transfer beyond the training environment. In this framework, mediation failures are informative, indicating that targeted mechanisms may be necessary but not sufficient for the desired functional change.

Therefore, we suggest generalization to the environment or “scaffolding,” as an additional candidate active ingredient. DCIs often improve task performance without transfer, suggesting that components explicitly designed to support real-world application may be essential. Examples include metacognitive coaching that links trained skills to everyday use, explicit action plans that specify when to deploy strategies, and/or bridging tasks that simulate real-world contexts [87,88]. These components can be tested orthogonally in dismantling designs—for example, comparing interventions with versus without strategy coaching. If such additions improve functional outcomes without increasing neural target engagement, this would indicate that scaffolding primarily drives generalization rather than core neuroplastic change.

In this way, mechanistic success without transfer becomes a guidepost rather than a failure, motivating a shift from intensifying training alone to incorporating components that support translation to real-world function.

Future directions should include the following:

- Larger, multisite trials integrating repeated RDoC-aligned mechanistic assessments to evaluate durability.
- Wearable or ecological momentary assessment tools for scalable, real-world measurement of cognitive and reward system engagement.
- Factorial dismantling designs to systematically test component interactions across RDoC domains.
- Precision neuroscience approaches using baseline neural, cognitive, and biological markers to predict which participants respond to specific intervention components.
- Modular DCIs in which specific DCI features can be manipulated while holding other features constant to enable systematic dismantling studies.

By “precision neuroscience,” we suggest that multimodal brain, behavior, and digital phenotyping data be collected in clinical trials of DCIs to identify which individuals benefit from which DCI components, through which mechanisms, and under what conditions. This approach goes beyond pre- or postimaging alone. A precision neuroscience framework may incorporate baseline and repeated neural measures, cognitive performance profiles, physiological signals, wearable data, ecological momentary assessment, and trial-level behavioral features to estimate individual response trajectories and adapt intervention delivery in real time. In this way, precision neuroscience can inform not only whether a component engages its target but also for whom, when, and at what dose it is most effective.

By explicitly mapping DCI components to RDoC constructs, domains, and units of analysis, future studies can generate mechanistically precise, scalable, and personalized interventions that target core processes driving psychiatric dysfunction. Furthermore, it may move the field away from simply demonstrating measurable neural change to drawing causal maps from manipulable components to target engagement to durable, generalizable outcomes that are capable of causal inference about why interventions fail and where they fail.

## Abbreviations

CoNECCT: Computerized Neuroplasticity Enhancing Cognitive Control Training
DCI: digital cognitive intervention
EEG: electroencephalography
ET: experimental therapeutics
fMRI: functional magnetic resonance imaging
MDD: major depressive disorder
NIMH: National Institute of Mental Health
RDoC: Research Domain Criteria
